# An Experimental Analysis of Five Household Equipment-Based Methods for Decontamination and Reuse of Surgical Masks

**DOI:** 10.3390/ijerph19063296

**Published:** 2022-03-11

**Authors:** Elena Scaglione, Gianluigi De Falco, Giuseppe Mantova, Valeria Caturano, Alessia Stornaiuolo, Andrea D’Anna, Paola Salvatore

**Affiliations:** 1 Dipartimento di Medicina Molecolare e Biotecnologie Mediche, Università degli Studi di Napoli Federico II, Via S. Pansini 5, 80131 Napoli, Italy; elena.scaglione@unina.it (E.S.); giuseppe.mantova@unina.it (G.M.); vale.caturano@gmail.com (V.C.); alestornaiuolo@gmail.com (A.S.); paola.salvatore@unina.it (P.S.); 2 Dipartimento di Ingegneria Chimica, dei Materiali e della Produzione Industriale, Università degli Studi di Napoli Federico II, Piazzale Tecchio, 80, 80125 Napoli, Italy; anddanna@unina.it; 3 CeSMA Centro Servizi Metrologici e Tecnologici Avanzati, Università degli Studi di Napoli Federico II, 80146 Napoli, Italy; 4 CEINGE, Biotecnologie Avanzate s.c.ar.l., Via G. Salvatore 436, 80131 Napoli, Italy; 5 Task Force on Microbiome Studies, Università degli Studi di Napoli Federico II, 80131Napoli, Italy

**Keywords:** reuse, type II surgical mask, household equipment, microbial cleaning, filtration efficiency, breathability

## Abstract

The current coronavirus pandemic has increased worldwide consumption of individual protective devices. Single-use surgical masks are one of the most used devices to prevent the transmission of the COVID-19 virus. Nevertheless, the improper management of such protective equipment threatens our environment with a new form of plastic pollution. With the intention of contributing to a responsible policy of recycling, in the present work, five decontamination methods for used surgical masks that can be easily replicated with common household equipment are described. The decontamination procedures were hot water at 40 °C and 80 °C; autoclave; microwave at 750 W; and ultraviolet germicidal irradiation. After each decontamination procedure, the bacterial load reduction of *Staphylococcus aureus* ATCC 6538 was recorded to verify the effectiveness of these methods and, moreover, bacterial filtration efficiency and breathability tests were performed to evaluate mask performances. The best results were obtained with the immersion in 80 °C water and the microwave-assisted sterilization. Both methods achieved a high degree of mask decontamination without altering the filtration efficiency and breathability, in accordance with the quality standard. The proposed decontamination methods represent a useful approach to reduce the environmental impact of this new waste material. Moreover, these procedures can be easily reproduced with common household equipment to increase the recycling efforts.

## 1. Introduction

The COVID-19 pandemic was caused by the community transmission of the SARS-CoV-2 virus (Severe Acute Respiratory Syndrome CoronaVirus 2), a virus ranging in size from 60–140 nm [1]. The main routes of infection transmission for coronaviruses are represented by respiratory droplets with dimensions between 5–10 µm and through aerosols containing viral particles with lower dimensions down to 50 nm [2,3], and they have been confirmed also for the SARS-CoV-2 virus [4,5,6]. The use of the surgical mask as a single-use protective device has become essential for the control of SARS-CoV-2 virus spread. Recent studies showed that about 40% of the respiratory droplets released by asymptomatic subjects who did not use a mask were positive for coronavirus. On the other hand, no coronavirus in either the aerosol or droplets was detected from an individual wearing a surgical mask, confirming how the use of a surgical mask counteracts the spread of the virus from infected individuals [7,8]. For these reasons, governments worldwide quickly mandated that wearing a mask was obligatory in public areas and on public transportation, as and in some cases, outdoors. Moreover, due to the concerning rise of new variants of SARS-CoV-2 coronavirus, it is likely that the production and the diffusion of personal protective equipment (PPE), and particularly face masks, will be an issue to deal with again in the near future.

It is now established that the use of PPE represents the first effective method to prevent infection. However, regarding face masks in the context of the COVID-19 pandemic, limited stores and production have prompted a widespread effort in evaluating both their extended use and reuse [9]. A recent report from the UK Foreign Commonwealth & Development Office (FCDO) has predicted that the demand for face masks will continue to increase even if the rollout of vaccination campaigns is considered [10]. The number of medical masks used is estimated to be in the order of 1 billion per month in Italy, corresponding to 66,000 tons/year of waste and a monthly consumption of 129 billion face masks worldwide [11].

Filtering face masks usually consist of up to six different layers containing plastic polymers such as polypropylene, polyurethane or polyacrylonitrile. They are classified based on their filtration efficiency into Filtering Face Piece Type 1 (FFP1, 80%), Filtering Face Piece Type 2 (FFP2, 94%) and Filtering Face Piece Type 3 (FFP3, 99%) in the UE and into Surgical N95 Respirators (N95, 95%) and Surgical N99 Respirators (N99, 99%) in the U.S. [12]. Surgical masks are categorized for filtration efficiency into Type I (≥95%), Type II (≥98%) and Type IIR (≥98%) (UNI EN 14683:2019). They are less effective when compared to respirators in terms of filtration efficiency and are composed of three layers of nonwoven materials, e.g., spunbond and meltblown fabrics containing polyethylene, polypropylene and polyethylene terephthalate [13]. Therefore, the rise in the consumption of filtering face masks and surgical masks and their incorrect disposal could result in a significant addition to plastic pollution, eventually threatening the health of oceans and marine life [14].

Although surgical masks have been designed to be single-use equipment, their safe reuse for a limited time could provide a reliable disposal alternative, thereby reducing the issues connected to waste production. For this reason, safe decontamination methods for on-site disinfection can be proposed with the aim of reducing the severe shortage of masks and their environmental and economic burdens [15,16]. During the first period of the pandemic emergency in April 2020, the Centers for Disease Control and Prevention (CDC) provided guidelines describing three processes for the de-contamination and reuse of filtering facepiece respirators based on ultraviolet (UV) germicidal irradiation, vaporous hydrogen peroxide, and moist heat, respectively [17]. The germicidal effect of UV light has been proven to have a negligible adverse effect on mask performance in terms of filtration efficiency and did not change the polymer structure, morphology or pressure [18]. The use of both dry heat and steam-generated heat has been successfully applied to decontaminate face masks without significantly altering the blocking efficacy [7,19]. Similarly, Andreola et al. [20] proposed a protocol based on hot household hygroscopic materials for the effective inactivation of viral infectivity on non-medical face mask surfaces. The use of supercritical CO_2_ for microorganisms’ inactivation was suggested to develop a protocol for cleaning and sterilization of FFP2 masks, preserving the filtration performances up to 10 cycles of application [21]. Charvet et al. investigated the impact of different washing procedures on both surgical and FFP2 masks, showing that the modifications of surface properties and fibrous structure after washing affected the performance of the masks, particularly for the filtration of submicronic particles [22].

The purpose of the present work is to describe and characterize experimental methods for surgical mask decontamination that can be easily replicated on household equipment. The methods herein presented are based on hot water at 40 °C and 80 °C, autoclave steam, microwave-generated steam and ultraviolet germicidal irradiation (UVGI). The decontamination methods proposed here did not affect the surgical masks’ performance, as demonstrated by Bacterial Filtration Efficiency (BFE) and Breathability (BRE) results. They were designed both to maintain the effectiveness of surgical masks and to be readily available to the community, providing a rational basis for implementing the reuse of surgical masks and improving the sustainability of single-use medical devices.

## 2. Materials and Methods

### 2.1. Bacterial Strain and Growth Conditions

The strain used in this study was *Staphylococcus aureus* ATCC 6538, a Gram-positive bacterium. This strain was chosen, as indicated by the UNI EN14683-2019 standard, as a reference to be used in the BFE tests for the characterization of surgical masks. Moreover, *S. aureus* is a pathogenic bacterium that causes respiratory diseases in the community, and it is also more resistant to different decontamination methods [23]. The microorganism was cultured in broth and agar media at 37 °C. The media used were Tryptic Soy Broth (TSB) and TS agar (TSA) (OXOID, Basingstoke, Hampshire, UK). The strain was stored frozen at −80 °C in TSB supplemented with 10% glycerol (*v/v*) (Carlo Erba, Reagents, Milan, Italy) until use and the working cultures were activated in the respective broth at 37 °C for 15–18 h [24].

### 2.2. Microbial Shedding and Cleaning of Single-Use Surgical Masks

In this study, *S. aureus* ATCC 6538 was cultured overnight in TSB at 37 °C, with shaking (200 rpm). *S. aureus* ATCC 6538 was diluted to 10^8^ Colony Forming Units (CFU)/mL in peptone water (Peptone 10 g/L, VWR International; NaCl 5 g/L, Carlo Erba Reagents, Milan, Italy). In order to simulate the condition of a mask after usage, commercially type-II surgical masks (GDA s.r.l., Galatina, Lecce, Italy), whose performances meet the standard UNI EN 14683:2019 requirements (BFE ≥ 98%; BRE < 40 Pa/cm^2^), were fully submerged into bacterial solution and subsequently placed on an orbital stirrer (Incubator shaker G25, New Brunswick scientific Co., Inc., Edison, NJ, USA) for 15 min at 200 rpm at room temperature. Finally, the masks were removed and allowed to dry in a biosafety cabinet (ESCO Class II, BSC) for approximately 1–1.5 h before proceeding with the decontamination treatments. In order to verify the *S. aureus* ATCC 6538 concentration on soiled surgical masks, a viable bacterial counting was performed. The whole masks were placed in a sterile flask containing 100 mL of extraction fluid (Peptone 1 g/L; NaCl 5 g/L; Polysorbate surfactant 2 g/L, Sigma-Aldrich, St. Louis, Missouri, USA). The flask was placed on an orbital stirrer for 15 min (200 rpm) at room temperature. Afterward, 100 mL of the extraction fluid was filtered through a 0.22 μm filter (Merck KGaA, Darmstadt, Germany). The filter was soaked in Phosphate-Buffered Saline (PBS) and serial dilutions were plated on TSA to determine the total microbial count [25].

### 2.3. Decontamination Methods

The following decontamination methods were investigated:

- Immersion in 40 °C hot water, 20 min for each cycle;
- Immersion in 80 °C hot water, 20 min for each cycle;
- Exposure to autoclave steam, 21 min at 121 °C and 1 atm for each cycle;
- Microwave-assisted sterilization, at 750 W for 2 min for each cycle;
- Exposure to ultraviolet germicidal irradiation (UVGI), at a distance of 18 cm for 30 min for each cycle.

To perform the immersion procedure in hot water, bi-distilled water was heated using a thermostatic bath (GFL 1083, Gesellschaft für Labor technik, Burgwedel, Germany ) up to 40 °C or 80 °C, and the soiled masks were fully submerged for 20 min for each cycle.

The treatment in autoclave (ORION-Pool Bioanalysis Italiana PBI) was performed by placing the soiled masks into heat-resistant bags for 21 min at 121 °C and 1 atm for each cycle.

The treatment with microwave-generated steam was performed by placing soiled masks without a plastic noseband inside off-the-shelf microwave steam bags with 50 mL of autoclaved water. The bags were then inserted into a commercial microwave (Samsung GE732K/XET) and treated at 750 W for 2 min for each cycle. At the end of each treatment, wet masks were allowed to dry in a biosafety cabinet for approximately 1.5 h.

For the UVGI treatment, the inner sides of the mask samples were exposed to UV light (Vilber Lourmat, 30 W-254 nm tube) at a distance of 18 cm for 30 min for each cycle.

In order to verify the *S. aureus* ATCC 6538 viable count after each decontamination method on soiled surgical masks, the devices underwent the extraction procedure and serial dilutions were plated on TSA as described above. All experiments were performed in duplicate with two independent experiments. The results obtained were analyzed and graphically reported and statistical significance was examined by the Student’s *t*-test.

### 2.4. Bacterial Filtration Efficiency and Breathability Tests

To evaluate the efficacy of the proposed different decontamination procedures on soiled masks, the BFE test (according to the standard UNI EN 14683:2019) and BRE tests (according to the standard UNI EN 14683:2019 and the standard UNI EN ISO 9237:1997) were performed using the commercially available system Bulldog Plus Bio (XEARPRO, Cogliate, Italy).

For the BFE measurements, the system is composed by an electronic sampler Bulldog Aero, an aerosol generator, that produces aerosol particles by nebulizing the bacterial solution, a pyrex and Teflon aerosol chamber, a glass condenser placed downstream of the impactor, an electronic flow sampler Bulldog Plus Bio and an Andersen-type six-stages cascade impactor (see Supplementary Figure S1).

Briefly, a constant bacterial aerosol containing 5 × 10^5^ CFU/mL of *S. aureus* ATCC 6538 is produced by the aerosol generator and then passed through the mask samples and through the 6-stage cascade impactor with a flow rate of 28.3 L/min, controlled by the electronic flow sampler. Each of the six stages consisted of 400 orifices and a petri dish containing the TSA medium that was used to collect the aerosolized sample. The cut-point size d_50_ of each stage is reported in Supplementary Table S1 [26]. The results of the clean and soiled masks were then compared to the negative control and positive control stream of *S. aureus* ATCC 6538. The BRE tests were performed by connecting the electronic flow sampler Bulldog Plus Bio to a base unit equipped with a sample holder. A constant air flow rate of 8 L/min is sent to an exposed area of the mask sample equal to 4.9 cm^2^ and the differential pressure is measured by the Bulldog Plus Bio system. For the clean surgical mask used as control (CTRL), BFE and BRE test results were equal to 100% and <40 Pa/cm^2^, respectively. All experiments were performed in duplicate with two independent experiments. The results obtained were analyzed and graphically reported and statistical significance was examined by a Student’s *t*-test.

## 3. Results and Discussion

**Figure 1:**
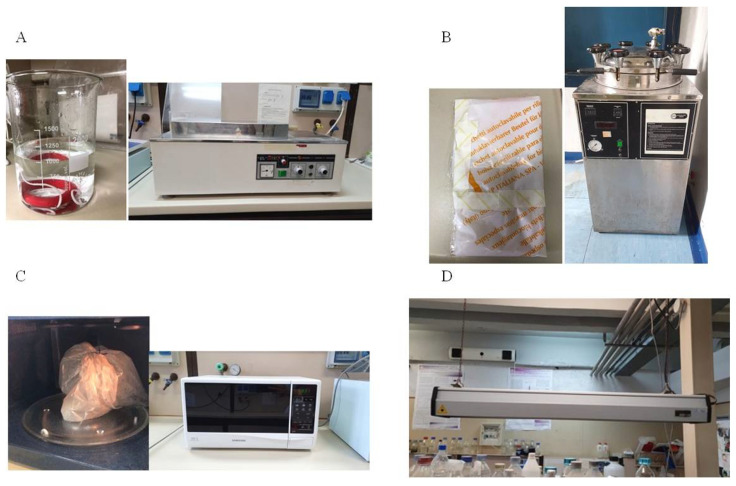
Pictures of the equipment used for the decontamination methods: immersion in hot water (**A**); exposure to autoclave steam (**B**); microwave-assisted sterilization (**C**); exposure to UVGI (**D**).

In the present work we evaluated the efficacy of five methods of decontamination of soiled single-use masks to reproduce in house settings (Figure 1). In detail, we analyzed: immersion in hot water at 40 °C (40 °C) and at 80 °C (80 °C) (Figure 1A); exposure to autoclave steam (AC) (Figure 1B); microwave-assisted sterilization (MW) (Figure 1C) and exposure to UVGI (UVGI) (Figure 1D). Briefly, single use surgical masks were soiled with a standardized bacterial suspension of *S. aureus* ATCC (10^8^ CFU/mL). After that, the extraction procedure of soiled masks confirmed the presence of 10^8^ CFU/mL bacterial cells on the devices (data not shown). After each procedure, the BFE and BRE tests were performed. In particular, the mean size of the bacterial aerosol used for the BFE measurements was derived from the number particle size distribution function, which was equal to 2.7 ± 0.1 µm.

**Figure 2:**
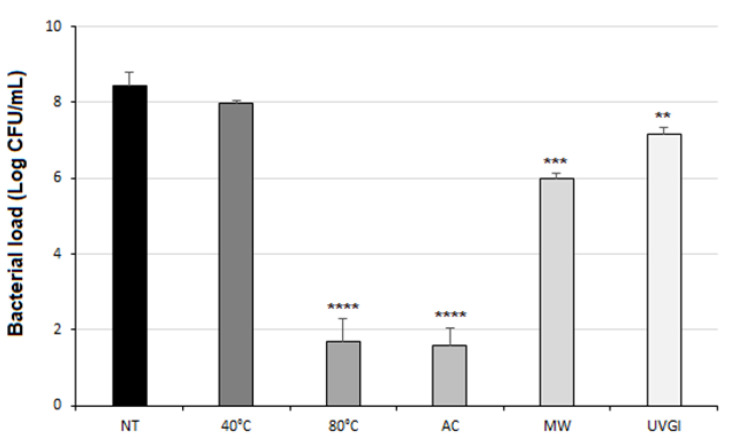
Bacterial load on soiled masks after one cycle of each decontamination method. Bacterial load (Log CFU/mL) on soiled masks after one cycle of each decontamination method. Not treated (NT); immersion in hot water at 40 °C (40 °C); immersion in hot water at 80 °C (80 °C); to autoclave steam (AC); microwave-assisted sterilization (MW) and exposure to UVGI (UVGI). Results are reported as mean ± SD of two independent experiments) (**, *p* < 0.01; ***, *p* < 0.001; ****, *p* < 0.0001).

After one cycle of each decontamination method, the bacterial load reduction was evaluated compared to that of untreated soiled masks (NT) (Figure 2). Compared to NT, the autoclave steam and immersion in hot water at 80 °C treatment drastically reduced by >6-Logs the bacterial load present on the treated soiled devices. These results are completely in accordance with the FDA specifications, which consider an adequate decontamination method as one that guarantees a reduction of the microbial load of at least 3-Logs [27,28].

### 3.1. Heat Treatments

Heat treatments, including dry heat and moist heat, decontaminate a mask by irreversible coagulation and denaturation of microbial or viral proteins, usually at a temperature above 70 °C [29]. The reduction of pathogens at a particular temperature is highly dependent on exposure time, matrix, and the microbial group [30].

The sterilization obtained through hot water immersion represents a method widely suitable in home settings as it is a non-toxic, cost-effective and rapid procedure for the deterioration of microorganisms. The polypropylene fibers in N95 masks have a thermal degradation point of 130 °C, so the heat treatment should not exceed this temperature [31]. Heat treatments for mask decontamination using temperatures between 70 °C and 80 °C are recommended [32].

The immersion in hot water at 40 °C has not led to a significant bacterial load reduction (Figure 2); as expected, bacteria are frequently subjected to shifts in temperature during their life cycle especially in human host [33]. Whereas both the BFE and the BRE tests highlighted no signs of functions deterioration after one cycle of decontamination at 40 °C (see Supplementary Figure S2). As this procedure didn’t determine an effective bacterial cleaning, no additional cycles at 40 °C were performed. Conversely, the same method performed with water temperature of 80 °C showed encouraging results. After one cycle of this treatment, indeed, the number of bacteria on soiled devices has been drastically reduced, as reported in Figure 2 (****, *p* < 0.0001).

**Figure 3:**
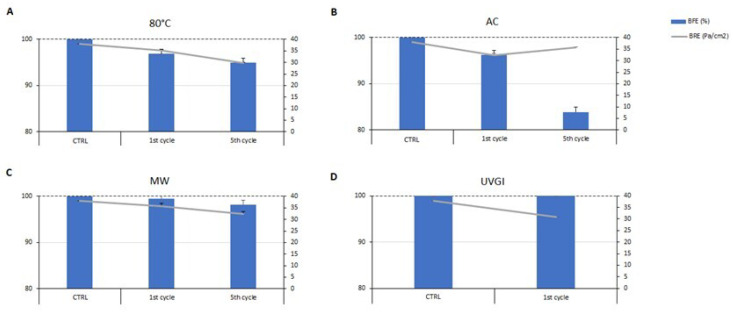
Evaluation of bacterial filtration efficiency (BFE, %) and breathability (BRE, Pa/cm^2^) post decontamination methods on a soiled single-use surgical mask after the first and fifth cycle of each decontamination method. (**A**) immersion in hot water at 80 °C (80 °C); (**B**) autoclave steam (AC); (**C**) microwave-assisted sterilization (MW); (**D**) ultraviolet germicidal irradiation (UVGI). CTRL, clean surgical mask. Results are reported as mean ± SD of two independent experiments.

Therefore, five cycles of decontamination have been performed on the same device to characterize its durability. The results obtained by BFE and BRE tests, reported in Figure 3, showed that the 80 °C water immersion decontamination method did not alter the breathability of the decontaminated surgical mask, even in subsequent cycles. Although the filtration efficiency was reduced to 95% after five cycles of immersion in 80 °C hot water, the mask could be re-used as a surgical mask type I (BFE ≥ 95%, UNI EN 14683:2019) (Figure 3A). It is worth highlighting that no conclusions can be drawn with regard to the effect of the treatments on the fibrous structure in a dimensional scale lower than the range of the bacterial aerosol used for BFE measurements.

Another physical approach used in the present work was that of an autoclave. It works on the principle of moist heat sterilization, wherein saturated steam is generated under pressure in order to kill microorganisms such as bacteria, viruses, and even heat-resistant endospores. This method has been explored since it can be easily performed by means of a pressure cooker in domestic settings, which provides an acceptable alternative to the autoclave sterilization [34]. The soiled single-use surgical masks decontaminated after one cycle of an autoclave exhibited a significant reduction of bacterial load on the mask surface of seven orders of magnitude (****, *p* < 0.0001) (Figure 2). Moreover, both BRE and BFE values reported in Figure 3 show that the autoclave steam treatment did not significantly affect the filtration efficiency and the breathability of the mask after one cycle, meaning that it was still suitable according to the standards. Conversely, the filtration efficiency decreased after five cycles of decontamination performed on the soiled masks, reaching a value of 83% (Figure 3). Therefore, this method cannot be applied for multiple cycles of decontamination. Nevertheless, the bacterial load reduction obtained after one cycle suggests that a single cycle of the autoclave is sufficient to achieve the complete decontamination of a single-use surgical mask. It is worthy of note that several studies suggest that the use of an autoclave is not recommended, particularly for N95 FFRs, given the significant increase in particle penetration and deformation [35,36]. Another method that is easy to reproduce in the home setting is represented by microwave-generated steam, a source of warm moist heat widely available. However, microwave ovens are not recommended because microwave irradiation may lead to fiber melting, although the use of a steam bag allows for the overcoming of such a limitation. Steam generation is typically achieved by the introduction of microwave steam bags containing water. Off-the-shelf microwave steam bags have been commercially available for decades, predominantly for the purposes of disinfecting breast pump and infant feeding accessories [37]. While the antimicrobial activity of microwave energy itself varies based on power and duration, and the steam produced by heating water with microwaves represents the rationale of the decontamination process, steam leads to the irreversible coagulation and denaturation of microbial proteins [29,38]. The use of steam and microwave-generated moist heat can be useful, as microwave ovens are commonly used in domestic settings, and the treatment time to obtain the inactivation of microorganisms is only 2 min [39]. However, this approach may only be suitable for families or small organizations, as a microwave oven can only be used to treat one mask at a time. Furthermore, users need to be mindful of the power delivered by their microwave oven and about the dimensions of the reservoir and volume of water used, as these will affect the steam production [40]. Moreover, it is recommended to not use the same microwave as one that is used for cooking. Finally, the nose metal strip of surgical masks can be a limitation for this decontamination method, leading to sparks when using the microwave oven. For that reason, the proposed procedure can be applied on commercial surgical masks without metal nose strips, ensuring safe decontamination.

The results obtained on soiled surgical masks after one cycle of a microwave-generated steam procedure showed a reduction of the microbial load of two orders of magnitude compared to untreated masks (***, *p* < 0.001), as reported in Figure 2. Furthermore, the BFE and BRE tests showed that the microwave-generated steam method did not alter either filtration efficiency or pressure drop of cleaned surgical masks, even after multiple cycles of decontamination (Figure 3). In fact, after five cycles, the filtration efficiency slightly decreases but still meets the quality standards required for Type II surgical masks.

Bergam et al. [41] employed three treatment cycles of microwave generated steam for decontaminating filtering facepiece respirators and found that particle penetration remained within acceptable limits even after multiple treatment applications [41]. Moreover, the use of a microwave for mask decontamination did not determine significant changes in fit, odor detection and comfort for the mask wearer [42].

### 3.2. Ultraviolet Germicidal Irradiation Treatment

UVGI involves the use of ultraviolet (UV) electromagnetic waves, particularly UVC in the wavelength range of 200–280 nm, in which photons are absorbed by viral or microbial nucleic acids and thereby damaging DNA/RNA and preventing replication [32]. UV light has historically been utilized in the disinfection and purification of air, water and food, and has recently become a regular component of medical sanitation systems [43]. Using UVGI as a method for mask decontamination requires the installation of a UVGI device, which can range in size and cost depending on the processing load. Since UVGI systems are already used in many healthcare facilities, it may be feasible and cost-effective to adapt biological safety cabinets or surface sterilization equipment to decontaminate surgical masks. In addition, a UVGI system can be installed for an entire room where many surgical masks can be decontaminated at the same time. Several researchers have pointed out that the UVGI dose is critical to the success of the method; an insufficient dose will not reach all internal surfaces and will leave active infectious material, while an overdose could compromise the structural integrity of the mask and reduce filtration performance [40].

One cycle of 30 min with light directed towards the inner layer of the mask was performed. The number of bacteria on the soiled surgical mask surface decreased by only one magnitude of order (**, *p* < 0.01) after the treatment (Figure 2). Moreover, the BFE and BRE tests showed that one cycle of decontamination did not alter the performance of the surgical mask, as reported in Figure 3.

Although the obtained data regarding antimicrobial efficiency and BRE and BFE values are promising, there are several disadvantages to UVGI treatment. Indeed, its effectiveness is largely dependent on the intensity of radiation and the length of exposure; in addition, most UV systems use low pressure mercury lamps, which entail the risk of mercury pollution and the release of ozone, with its consequent toxic effects [39]. For these reasons, and since the bacteria load reduction was lower than the other decontamination methods, no additional cycles were performed.

## 4. Conclusions

The strong environmental impact resulting from the massive use of PPE requires the development of methods for surgical mask decontamination and reuse. Although these devices are not designed to be reused [36], the decontamination methods must guarantee few requirements, such as microbial cleaning, bacterial filtration efficiency and breathability [44,45]. To analyze the efficiency of the decontamination methods, in the present work, we simulated the bacterial contamination of single-use type II surgical masks and then evaluated the microbial cleaning after each proposed treatment. Following one cycle of the decontamination methods tested, a significant bacterial reduction was recorded after the immersion of a soiled surgical mask in 80 °C water. Even after five cycles, the values of bacterial filtration efficiency and breathability meet the quality standards for a type I surgical mask, suggesting a possible safety reuse of the device with an easy-to-use method in home settings. It is worth noting that the use of surgical mask type I is still recommended by the World Health Organization as a way to counteract COVID-19 transmission [46]. Likewise, a substantial bacterial load reduction was achieved by placing soiled surgical masks into an autoclave for one cycle. Moreover, the mask performances were not affected by a single cycle of autoclave sterilization. However, this method cannot be used for multiple cycles of decontamination, since the filtration efficiency significantly decreased with subsequent cycles. A microwave-assisted sterilization method was performed using microwave steam bags containing soiled surgical masks soaked in 50 mL of water. The bacterial load recovered after one cycle of treatment decreased compared to untreated masks. Additionally, the method didn’t significantly alter either the bacterial filtration efficiency or the breathability with up to five cycles of application. Finally, a non-heat method was evaluated by exposing soiled surgical masks to UVGI. Even if one cycle of 30 min did not modify the mask features, the bacterial load recorded was not significantly reduced. For these reasons, this procedure was not further investigated, as it is more complicated to reproduce in a common household setting compared to the other methods evaluated in the present work. Although this study evaluated the efficiency of the decontamination methods on masks soiled with a bacterial strain, different literature data have demonstrated how all proposed methods have an effect on the viability of viruses and on other biological contaminants as well [47,48]. Moreover, the proposed methods are planned for domestic decontamination. Literature data suggests that the user’s normal flora is the main source of contamination of a surgical mask. Since the decontaminated surgical mask is reused by the same user, microorganisms that are still present on the decontaminated mask are not clinically concerning [49].

Taking into account the cost and availability of the necessary resources and equipment available in a domestic setting, the proposed decontamination methods could be considered safe and easy to reproduce, allowing one to reduce the environmental impact arising from the massive use of single-use surgical masks.

## Data Availability

The data presented in this study are available on request from the corresponding author.

## Supplementary Materials

The following supporting information can be downloaded at: https://www.mdpi.com/article/10.3390/ijerph19063296/s1, Figure S1: Bulldog Plus Bio system configuration for BFE analysis; Figure S2: Evaluation of Bacterial Filtration efficiency (BFE) (%) and Breathability (BRE) (Pa/cm^2^) after one cycle of water immersion at 40 °C; Table S1: Cut-point sizes of the Andersen impactor.
