# Supplementary material for: An Experimental Analysis of Five Household Equipment-Based Methods for Decontamination and Reuse of Surgical Masks
Source: Int J Environ Res Public Health. 2022 Mar 11;19(6):3296. doi: 10.3390/ijerph19063296 (PMC8952502; doi:10.3390/ijerph19063296)
Supplement: Supplementary file 1 [file ijerph-19-03296-s001.zip › ijerph-1604209-supplementary.pdf]

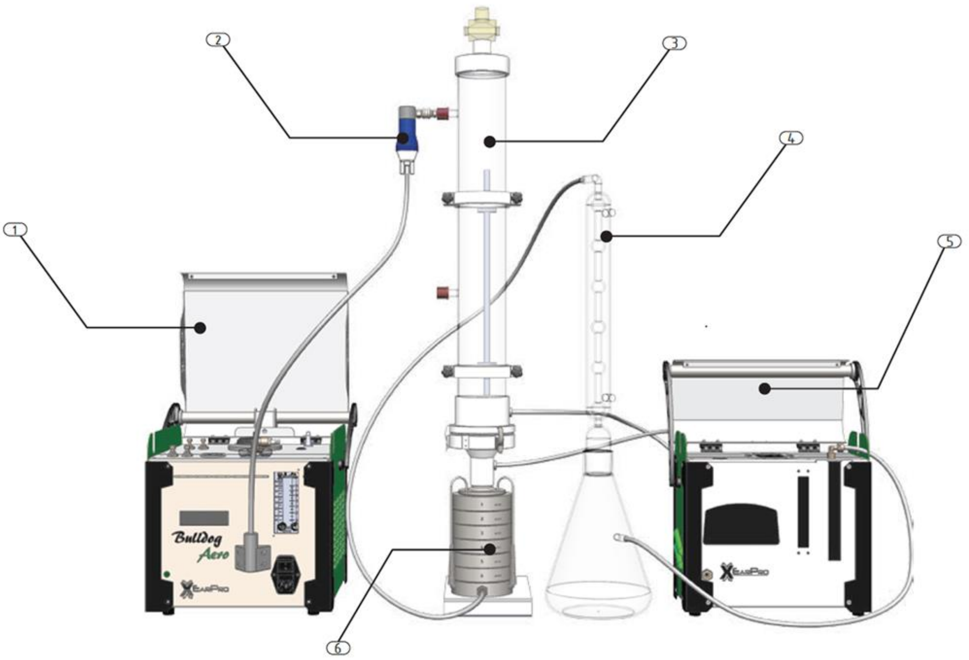

Supplemental Figure S1. Bulldog Plus Bio system configuration for BFE analysis: electronic sampler Bulldog Aero (1), an aerosol generator, that produces aerosol particles by nebulizing the bacterial solution (2), a pyrex and Teflon aerosol chamber (3), a glass condenser placed downstream of the impactor (4), an electronic flow sampler Bulldog Plus Bio (5) and an Andersen-type six-stages cascade impactor (6).

Supplemental Table S1. Cut-point sizes of the Andersen impactor.

Stage	D_{50}, µm
1	7.1
2	4.7
3	3.3
4	2.1
5	1.1
6	0.65

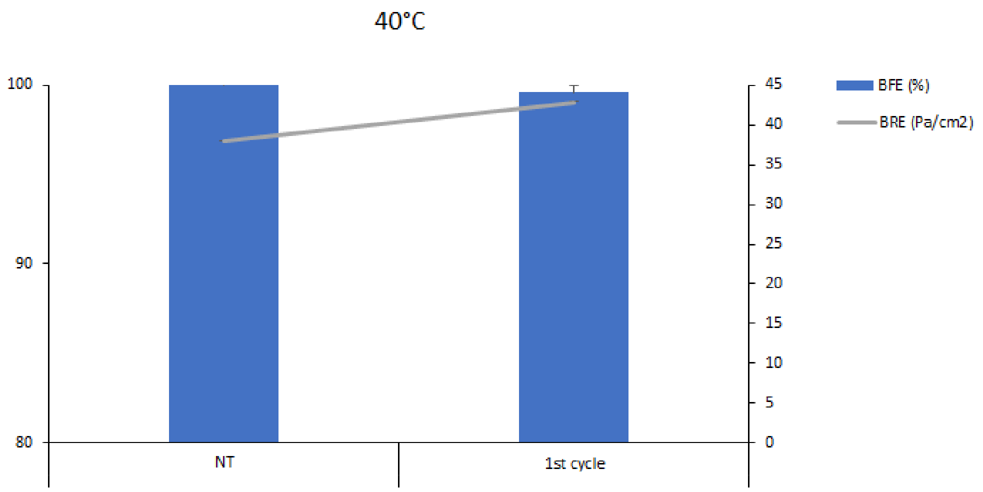

Supplemental Figure S2. Evaluation of Bacterial Filtration efficiency (BFE) (%) and Breathability (BRE) (Pa/cm^2) after one cycle of water immersion at 40 °C.
